# Chitosan-Coated Alginate Matrices with Protein-Based Biostimulants: A Controlled-Release System for Sustainable Agriculture

**DOI:** 10.3390/ma18030591

**Published:** 2025-01-28

**Authors:** Daniel Szopa, Katarzyna Pstrowska, Anna Witek-Krowiak

**Affiliations:** 1 Department of Chemical Process Engineering and Technology, Faculty of Chemistry, Wrocław University of Science and Technology, Gdańska 7/9, 50-344 Wrocław, Poland; daniel.szopa@pwr.edu.pl; 2 Department of Advanced Material Technologies, Faculty of Chemistry, Wrocław University of Science and Technology, Gdańska 7/9, 50-344 Wrocław, Poland; katarzyna.pstrowska@pwr.edu.pl

**Keywords:** hydrolysate, fertilizer, biopolymer, matrix, chitosan, alginate, coating

## Abstract

Developing biodegradable complex fertilizers is crucial for sustainable agriculture to reduce the environmental impact of mineral fertilizers and enhance soil quality. This study evaluated chitosan-based hydrogel coatings for sodium alginate matrices encapsulating amino acid hydrolysates from mealworm larvae, known for their plant growth-promoting properties. The research aims to identify the potential of biopolymer matrices for producing biodegradable slow-release fertilizers and to outline future development pathways necessary for this technology to be usable in the fertilizer industry. Chitosan coatings prepared with citric acid and crosslinked with ascorbic acid optimized plant growth, while those using acetic acid negatively affected it. Water absorption and nutrient release tests showed that chitosan coatings reduced water uptake and slowed initial nutrient release compared to uncoated samples. Leaching assays confirmed controlled-release behavior, with an initial burst followed by stability, driven by alginate–chitosan interactions and ion exchange. The X-ray diffraction (XRD) analysis revealed that adding hydrolysate and chitosan increased amorphousness and reduced porosity, improving structural properties. Thermogravimetric analysis (TGA) and Fourier-transform infrared (FTIR) spectroscopy demonstrated enhanced homogeneity and the presence of chemical interactions, which led to improvements in the material’s thermal stability and chemical characteristics. Biodegradation tests indicated greater durability of chitosan-coated composites, although hydrolysate incorporation accelerated decomposition due to its acidic pH. Germination tests confirmed no phytotoxicity and highlighted the potential of biopolymeric matrices for slow nutrient release. These findings indicate the possibilities of chitosan-coated alginate matrices as sustainable fertilizers, emphasizing the importance of adjusting coating composition and hydrolysate pH for enhanced efficacy and environmental benefits. The main recommendation for future research focuses on optimizing the chitosan coating process by exploring whether adding hydrolysate to the chitosan solution can reduce diffusional losses. Additionally, investigating the use of glycerol in the alginate matrix to minimize pore size and subsequent losses during coating is suggested. Future studies should prioritize analyzing percentage losses during the crosslinking of the alginate matrix, chitosan coating, and final shell crosslinking. This pioneering research highlights the potential for encapsulating liquid fertilizers in biopolymer matrices, offering promising applications in modern sustainable agriculture, which has not been studied in other publications.

## 1. Introduction

In the face of a growing global population and the need to ensure sufficient food supply, fertilizers have become an essential element of modern agriculture. Their use allows for increased crop yields, which is crucial for addressing global food security challenges. Projections indicate that global fertilizer consumption continues to rise—it increased by 4% in the financial year 2023 and is expected to reach 203.7 million tons of nutrients in 2024 [1]. However, traditional fertilizers pose significant environmental and resource-related challenges. Their production requires large quantities of non-renewable resources, such as natural gas, phosphate rock, and potash, as well as energy-intensive and costly technological processes. Furthermore, excessive and imprecise application leads to greenhouse gas emissions and nutrient leaching into surface waters. This results in the eutrophication of water bodies, ecosystem degradation, and the need for repeated fertilization due to the low retention of nutrients in the soil. The increasing use of fertilizers requires the adoption of modern, sustainable solutions. It is essential to utilize renewable resources and biopolymers in fertilizer production to reduce dependence on non-renewable resources and minimize the carbon footprint. Slow-release fertilizers (SRF) and controlled-release fertilizers (CRF), particularly those based on biodegradable coatings, enable the precise delivery of nutrients, minimizing environmental losses and supporting sustainable agriculture [2]. These fertilizers can also serve as carriers for encapsulating active ingredients such as fertilizers, pesticides, herbicides, and other agricultural agents [3].

Plant growth biostimulants are gaining growing interest in agriculture. Protein-based biostimulants derived from animal waste (such as fish, meat, and larvae) and plant residues (e.g., legume seeds) present a promising solution for modern agriculture. These biostimulants can be obtained through chemical (chemical hydrolysis) or enzymatic processing, resulting in hydrolysates rich in amino acids and short peptides. Such hydrolysates promote plant growth by increasing chlorophyll content, enhancing carbon uptake, and regulating metabolism, thereby improving seed germination, root development, nutrient absorption, and stress resistance. Additionally, they positively impact soil fertility and microbial activity [4]. It has been proven that biostimulants derived from *Tenebrio molitor* larvae exhibit beneficial properties for plant growth through chemical hydrolysis using acids and bases [5,6]. The publication by Szopa et al., (2024) [5] examined the influence of various extraction media, specifically individual acids and KOH, on the composition of hydrolysates in terms of macroelement content and their impact on the amino acid profile. The study also investigated the effect of the neutralization process on hydrolysate composition, where all hydrolysates were neutralized with a KOH-based hydrolysate and subsequently tested on plants in germination and pot experiments. The results demonstrated that the most effective hydrolysate was derived using H_2_SO_4_ at a concentration of 20%. Consequently, this hydrolysate was incorporated into matrix solutions designed to enable a slower release of the fertilizing substance. This approach was adopted because higher fertilizer doses were found to inhibit plant growth.

Biostimulants can be encapsulated using natural biopolymers by embedding them in a matrix through ionotropic gelation [7]. This method is relatively fast, does not require toxic reagents, and produces relatively large particles that are easy to use in agricultural applications. Natural polysaccharides such as alginate [8] or chitosan [9] can be used to produce polymer matrices. To further slow the release of active ingredients from hydrogel matrices, various coatings can be applied as an additional barrier to prevent the rapid leakage of ingredients. These coatings regulate the release rate of active compounds and are made from materials like biodegradable polymers (e.g., chitosan [10,11], cellulose [12], and PLA [13]), which modulate nutrient release based on environmental conditions such as soil moisture or pH.

This study aims to develop and evaluate the applicability of a chitosan-based hydrogel coating applied to sodium alginate matrices encapsulating amino acid hydrolysates. In the existing literature, hydrogel matrices have not been used to encapsulate liquid fertilizer compounds for fertilization purposes. They are typically applied to encapsulate solid microelement salts or microorganisms, highlighting a gap in this area [14,15,16]. The research question in the ongoing studies is whether it is possible to develop a biopolymer composite capable of encapsulating a liquid biostimulant within an alginate matrix. Additionally, it was crucial to resolve whether using such a matrix would allow for higher fertilizer doses and, thus, a better fertilization effect than the direct application. These hydrolysates were obtained through the chemical hydrolysis of high-protein material, specifically mealworm larvae. Given that these amino acid hydrolysates demonstrated plant growth-stimulating properties, encapsulation within a hydrogel matrix was proposed to enable controlled and prolonged release, as their effectiveness is maximized in small, sustained doses [5,6]. However, the alginate matrix alone resulted in excessively rapid hydrolysate release, creating challenges for storage and application. Chitosan is widely used in combination with sodium alginate due to its ionic interactions, but the novelty of this research lies in validating whether it is feasible to produce layered biopolymer fertilizers containing encapsulated liquid nutrients. This approach could lay the foundation for developing advanced solutions capable of encapsulating a variety of nutrients, beneficial bacteria, and other bioactive substances within layered biopolymer matrices, paving the way for complex and multifunctional fertilizer systems. Chitosan, through its interaction with alginate, is expected to reduce diffusion with the surrounding environment while enhancing the mechanical properties of the resulting composite. To confirm these properties, time-release and extraction tests were conducted to evaluate the controlled release of the hydrolysate and its bioavailability to plants. Morphological tests were performed to assess the impact of the chitosan coating and the encapsulated hydrolysate on capsule properties. Additionally, biodegradation tests of the biocomposites in soil were carried out.

## 2. Materials and Methods

In this study, sodium alginate, bentonite, and starch were used to create a biopolymer matrix for encapsulating liquid biostimulants. Chitosan, combined with ascorbic acid, was applied to coat the obtained alginate matrices containing the encapsulated hydrolysate. The matrix incorporated liquid biostimulants derived from dried, commercially available mealworm larvae (*Tenebrio molitor*) purchased from a pet store in Poland (Zooplus, Krakow, Poland). The biostimulants were produced through chemical hydrolysis using sulfuric acid (60% *m*/*m*) and neutralized with an alkaline hydrolysate based on potassium hydroxide (7.5% *m*/*m*), as described by Szopa et al., (2023) [6]. All chemical reagents were sourced from Chempur, Poland.

### 2.1. Preparation of Hydrolysates

Hydrolysates enclosed in a biopolymer matrix were prepared following the methodology described in a previous publication [6]. The material for chemical hydrolysis was mealworm larvae, subjected to acid hydrolysis with the use of H_2_SO_4_. Produced acid hydrolysate was then neutralized with an alkaline hydrolysate based on KOH. The neutralized hydrolysate were subjected to separation processes, where the liquid phase was retained as a biostimulant for encapsulation processes, and the solid phase was treated as waste.

### 2.2. Preparation of Biopolymer Matrix

**Figure 1:**
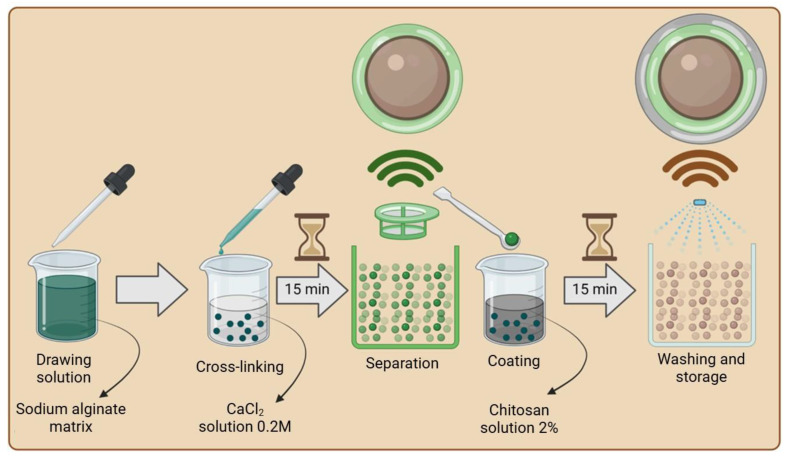
Scheme of alginate matrix with hydrolysate (AH) production (created with Biorender.com).

To prepare the AH (alginate hydrogel with immobilized hydrolysate) biopolymer matrix (Figure 1), a 4% *w*/*w* solution of sodium alginate was prepared, to which a neutralized hydrolysate with a pH of approximately 5.0 was added in a 1:1 mass ratio. Based on the total mass of the hydrolysate and biopolymer, matrix additives were incorporated into the solution: starch at 2% *w*/*w* and bentonite at 6% *w*/*w*. The mixture was heated to approximately 40 °C and stirred intensively until a homogeneous consistency was achieved, then cooled to room temperature. The resulting mixture was introduced into a crosslinking solution using a pipette. Due to the properties of sodium alginate, a 0.2 M CaCl_2_ solution was used as the crosslinking agent, which is commonly employed for ionic crosslinking during the encapsulation of microorganisms [17]. The crosslinking process lasted approximately 15 min, after which the resulting structures were separated using a sieve and rinsed with distilled water to remove excess calcium ions.

**Figure 2:**
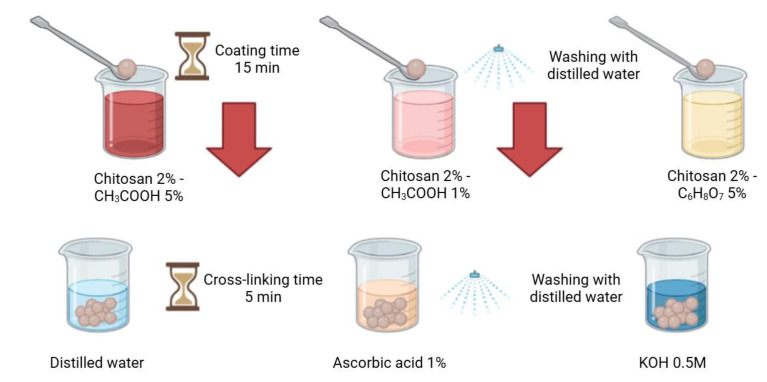
Scheme of different methods of applying chitosan (created with Biorender.com).

The next step involved coating the capsules with a chitosan layer (Figure 2). For this purpose, a 2% *w*/*w* solution of chitosan dissolved in either 1% or 5% *w*/*w* acetic acid or citric acid was prepared, depending on the tested coating method. The crosslinked AH composites were placed in the prepared chitosan solution, stirred for approximately 30 s, and left for 15 min to complete the coating process. Excess chitosan was removed by draining, and the composites were subsequently immersed (depending on the crosslinking method) in a 1% *w*/*w* ascorbic acid solution or a KOH solution for 5 min, or rinsed with distilled water. Finally, the crosslinked structures were thoroughly drained and rinsed with distilled water to remove any remaining residues. Composites A and C were prepared in the same manner, with the difference being that in the case of composite C, distilled water was used instead of the hydrolysate at pH 5.0.

### 2.3. Swelling Properties

The properties of the obtained structures were analyzed following drying and rehydration under simulated conditions. This study aimed to evaluate whether the developed structures could act as water reservoirs during drought and facilitate the release of nutrients from the matrix into the surrounding environment. For the investigation, 1 g of dried hydrogels was placed into 50 mL of a 1% *m*/*m* NaNO_3_ solution. Samples were taken at specific time intervals of 0.5, 1, 2, 4, 6, 24, and 48 h, after which the phases were separated. The hydrated structures were weighed to measure the increase in mass relative to their initial weight and then returned to the NaNO_3_ solution to monitor further swelling behavior. The liquid phase was subjected to elemental analysis to quantify the release of hydrolysate from the rehydrated matrix. The results were processed through statistical analysis to identify differences between the individual hydrogel samples.

### 2.4. Leaching Properties

Nutrient leaching under simulated soil conditions was evaluated using a glass column system. Throughout the week, a 1% *m*/*m* NaNO_3_ solution was continuously supplied to the column. The setup included a bottom layer of 0.15 g of glass wool, 2.5 g of porous filling, and a mixture of 5 g of ground soil with 2 g of fresh, crosslinked hydrogels. An additional 2.5 g of porous filling was placed on top. The NaNO_3_ solution was delivered from above using a peristaltic pump. Samples measuring 15 mL were collected at predetermined intervals over the 7 days. After completion, the collected samples were analyzed for elemental composition and subjected to statistical analysis to assess the dynamics of nutrient leaching under the simulated soil conditions.

### 2.5. Biodegradability

The biodegradability of the obtained biopolymer matrix was assessed over 28 days. To conduct the test, 2 g of the dried hydrogel matrix was placed inside a cotton bag, securely tied, and embedded in 20 g of soil. The soil was then moistened with distilled water until fully saturated and kept at ambient temperature for 48 h. After this initial incubation, the cotton bag was carefully removed, and the matrix capsules were placed on absorbent paper to eliminate any residual water before weighing. This procedure was repeated after 96 h and at four-week intervals. The soil was re-moistened every two days to maintain a consistently humid environment. All recorded data were analyzed using statistical methods.

### 2.6. ICP Analysis

Elemental composition analysis was conducted using an ICP-OES spectrometer (Varian, Australia). The procedure for sample preparation and analytical steps followed the methodology outlined by Szopa et al., (2023) [6].

### 2.7. XRD Analysis

The XRD analyses were performed using a MiniFlex diffractometer (Rigaku, Tokyo, Japan) equipped with a Cu anticathode (λ = 1.54178 Å). The diffractograms were collected from 5 to 90° with a speed of 0.09°/min.

### 2.8. TGA Analysis

The thermogravimetric analysis (TGA) was performed using a 5E-MAC6710 apparatus by CKIC (Changsha, Hunan, China). The changes in sample mass vs. temperature were registered under constant nitrogen flow during the temperature increase from 25 to 900 °C with a rate of 5 °C/min.

### 2.9. FTIR Analysis

The FTIR spectra were obtained using an IRAffinity-1S apparatus (Shimadzu, Kyoto, Japan) equipped with ATR Specac Quest (diamond crystal), in the range of 4000–400 cm^−1^, 256 scans.

### 2.10. Germination Tests

Germination tests were conducted to assess the potential phytotoxicity of the biocomposites. Four composites were examined: the first was control group A, containing the essential components of the alginate matrix; the second was control group C, which consisted of the same components as matrix A but was coated with a chitosan layer. The main tested groups were AH, where distilled water (used in group A) was replaced with a hydrolysate at pH 5.0 based on H_2_SO_4_, and CH, which was further enriched with a chitosan coating, similar to group C.

For groups AH and CH, biocomposite dosages were tested at 20%, 50%, 100%, 150%, and 200%. The germination period lasted 10 days, with fertilization applied on the 3rd day. All tests were conducted under sterile conditions, with the temperature maintained at 20 °C and humidity at 50%. The samples were watered every two days to avoid overflow and to monitor water retention properties. A soilless medium was used to eliminate microbial interference, ensuring a precise evaluation of dosage effects on the early stages of plant growth. After 10 days, biometric parameters were measured, including stem and root length, root surface area, and volume, along with fresh and dry biomass analysis. Subsequently, dried samples underwent elemental analysis to determine nutrient migration levels.

## 3. Results and Discussion

### 3.1. Evaluation of the Coating Method

**Figure 3:**
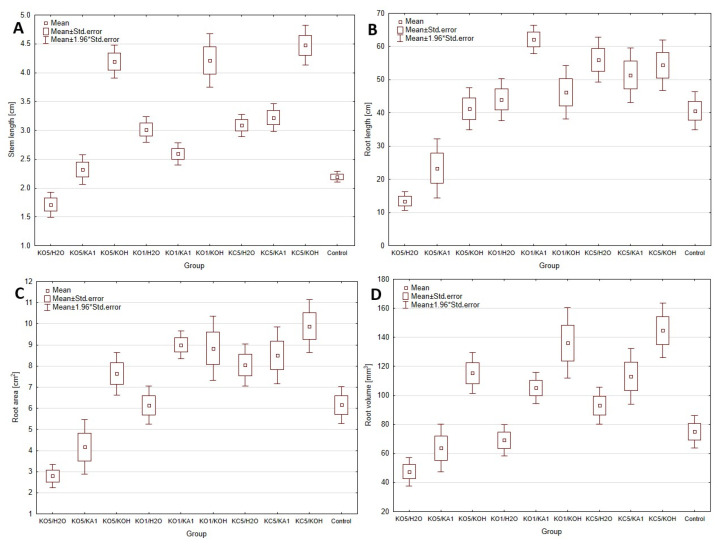
Biometric parameters of germination tests for types of coating, ((**A**)—Stem length analysis; (**B**)—Root length analysis; (**C**)—Root area analysis; (**D**)—Root volume analysis; * indicates *p* < 0.05 compared to the control group).

A key element in verifying the effectiveness of the tested chitosan coating methods was the analysis of their impact on plants, which was assessed through germination tests and the structure of the obtained composites. Figure 3 shows that the best plant growth results were achieved for composites crosslinked with a KOH solution, regardless of the solvent used for dissolving chitosan. However, the structure of these composites proved to be highly unstable and quickly degraded. The enhanced plant growth likely resulted from the additional K⁺ ions, which promote plant development [18]. The most favorable results were obtained for 5% *w*/*w* citric acid, used as a solvent to prepare a 2% *w*/*w* chitosan solution. On the contrary, the use of acetic acid adversely affected plant development. This is particularly intriguing, as 5% *w*/*w* acetic acid is the most commonly used solvent in the literature [19]. The adverse effects of this acid on plant growth are also confirmed by studies presented in Szopa et al., (2024) [5], where hydrolysate based on acetic acid, even at low doses, inhibited growth and, in some cases, caused plant death. The differences between chitosan crosslinking methods using distilled water and 1% *w*/*w* ascorbic acid were minimal. However, higher growth parameters, including increased root and shoot lengths, were observed with ascorbic acid. Therefore, subsequent research adopted the use of chitosan dissolved in citric acid and crosslinked with ascorbic acid as the preferred method for coating alginate matrices.

#### 3.1.1. Swelling Properties

**Figure 4:**
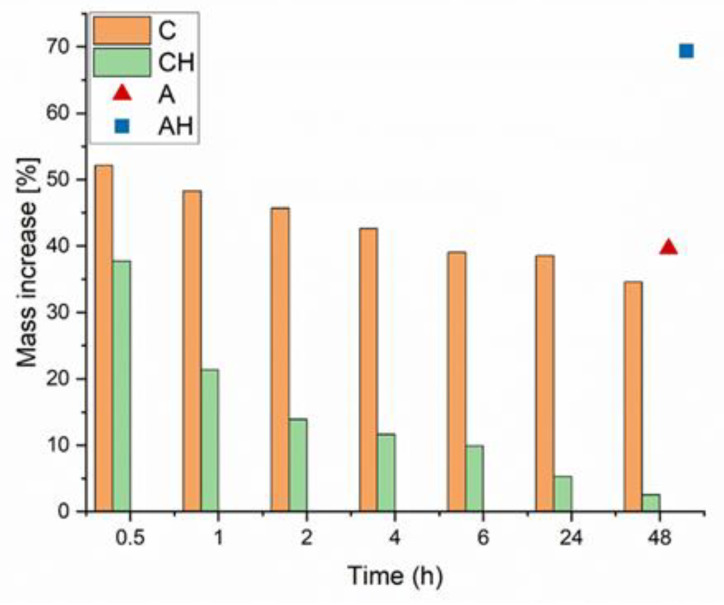
Change in mass over time due to swelling of dried hydrogels.

Water absorption tests were conducted on dried alginate composites coated with chitosan to evaluate their potential as water reservoirs in soil. Considering the established water absorption characteristics of chitosan, it was hypothesized that coating would improve the adsorption capacity of the resultant structures and facilitate the re-release of encapsulated fertilizer compounds [20]. The tests were performed on dried composites to simulate water re-absorption under drought-like conditions and assess whether a greater amount of hydrolysate was retained in the coated composite (CH) than uncoated composites (AH). The properties of the coated composites were compared to those of alginate composites without the chitosan coating after 48 h. Changes in composite mass over the time interval from 0.5 h to 48 h are presented in Figure 4.

It was observed that over time, the mass of composite C decreased, likely due to structural instability and/or partial degradation, which ultimately resulted in a mass increase of approximately 34%. A similar result of around 40% was obtained for the uncoated matrix A, but with a consistently upward trend, suggesting no structural degradation. In the case of composites containing hydrolysate, it was noted that the initial mass increase for the coated matrix was lower, followed by a sudden drop to only 2% (CH). Conversely, an opposite trend was observed for the AH sample: an initial increase of approximately 43%, followed by a linear rise to about 69% after 48 h. Differences in water absorption results may arise from the interaction of chitosan with the hydrolysate at a slightly acidic pH (5.0). Chitosan, at lower pH, increases its solubility due to protonation of amino groups (-NH_2_), which may weaken the biopolymer structure [21,22]. The reduced pH can also weaken the electrostatic bonds between sodium alginate and chitosan, as both polymers are sensitive to pH changes. It can, therefore, be assumed that the encapsulation of hydrolysate with a slightly acidic pH caused the protonation of carboxyl groups (-COOH), directly increasing the composite’s water absorption properties by enhancing porosity and enabling the formation of hydrogen bonds with water molecules [23].

The experiment suggests that alginate coatings are effective primarily for encapsulating liquid fertilizers with neutral or slightly alkaline pH, minimizing adverse interactions between chitosan, hydrolysate, and sodium alginate. To preserve the absorption properties crucial for fertilizer biocomposites, alternatives such as carboxymethyl cellulose (CMC) or pectin are suggested, as they are expected to interact better with sodium alginate under acidic conditions [24].

**Figure 5:**
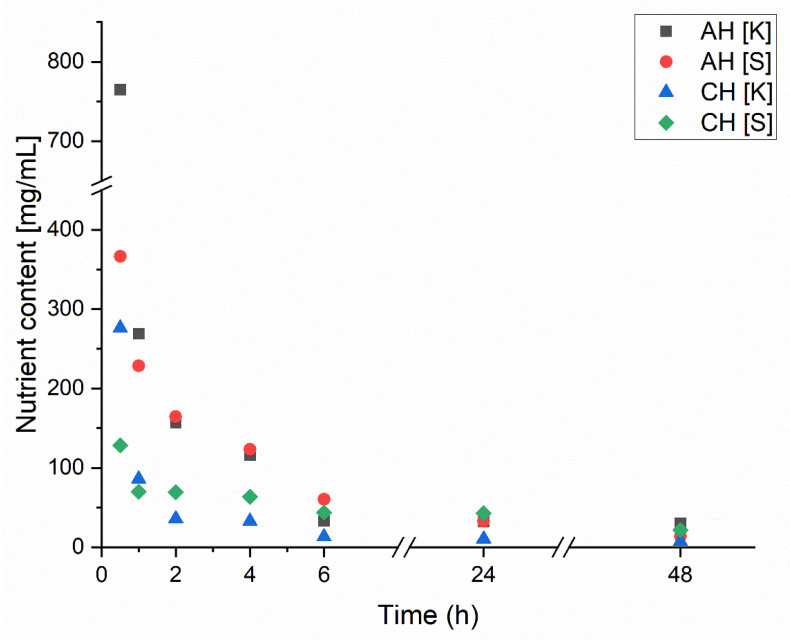
Change in nutrient release over time due to swelling of dried hydrogels.

The release of macronutrients during the swelling of dried composites was also evaluated to determine their capacity to re-release encapsulated nutrients and compare the results for coated (CH) and uncoated (AH) samples. The results, shown in Figure 5, present the release rate of two primary macronutrients contained in the hydrolysate: sulfur (S) and potassium (K). Macronutrient release was considerably higher for the uncoated samples (AH) than for the coated samples (CH). In the AH composites, the release transitioned to a linear and low rate after 6 h, maintaining this trend for the remaining 48 h for both nutrients. The differences in initial nutrient amounts within the composites result from the composition of the encapsulated hydrolysate. In the CH composite, most nutrients were released within approximately one hour, followed by a flat, linear trend only observed in the AH samples after 6 h. Additionally, the initial concentrations in the CH composite were 2–4 times lower than in the AH composite, which may indicate lower encapsulation efficiency for the hydrolysate in the coated samples. This observation is further supported by the rapid approach to near-zero nutrient concentrations in the later stages of swelling. Such rapid release excludes achieving a slow, sustained release of hydrolysate from CH composites. Otherwise, the nutrient release would have remained steady over a prolonged period before gradually decreasing. This phenomenon is intriguing because chitosan is known to form dense and less porous structures and, through electrostatic interactions with sodium alginate, should theoretically reduce composite porosity while retaining a more significant amount of hydrolysate. However, the slightly acidic pH of the encapsulated hydrolysate may have adversely affected the structure and stability of the CH composites [25]. It is also possible that excessive release of the hydrolysate occurred during the coating process in the AH composite. The lower pH weakened the crosslinking between sodium alginate and chitosan due to the protonation of amino groups, causing some hydrolysate to leak out [26]. This suggests that further research should be conducted using hydrolysates with a pH closer to neutral or by completely changing the polymer to improve encapsulation efficiency. While chitosan demonstrates good binding properties with alginate, it may only be suitable under specific conditions; otherwise, it will lose its mechanical properties and the functionality of its amino groups [27]. The results presented in Figure 5 indicate that excessive release of chitosan into the solution occurred during the encapsulation process. To address this issue, a potential solution to minimize the release of hydrolysate from the alginate matrix into the chitosan solution could involve limiting diffusion processes. This could be achieved by adding the encapsulated hydrolysate to the chitosan solution to equalize concentrations and thereby reduce release. Another approach involves incorporating glycerol into the alginate matrix. Acting as a plasticizer, glycerol densifies the alginate structure, effectively reducing porosity [28,29]. Through its interaction with the hydroxyl groups in the alginate chains, glycerol increases the mobility of these chains, promoting improved crosslinking with Ca^2^⁺ ions and subsequently reducing the number of pores. Additionally, it is recommended to use a hydrolysate with a more neutral pH of approximately 7.0. A neutral pH would mitigate the effects of an acidic environment, which can destabilize the alginate structure and increase porosity. This impact is evident in the comparison of the swelling properties of the A and AH matrices, as shown in Figure 4.

#### 3.1.2. Leaching Properties

**Figure 6:**
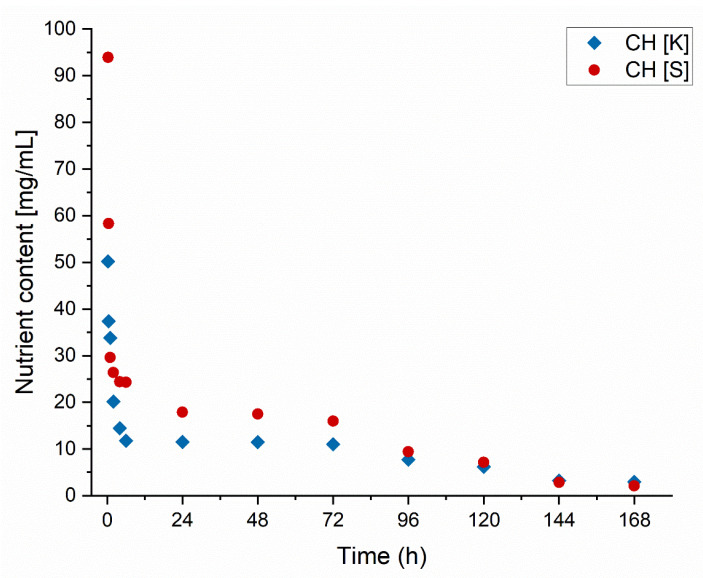
The amount of liquid leached out over time.

The leaching of nutrients was conducted for freshly crosslinked composites in a column through which a 1% *w*/*w* NaNO_3_ solution, simulating soil conditions, was passed. The release rate of encapsulated macronutrients in coated matrices was demonstrated, allowing for the determination of release dynamics and the duration of nutrient availability for plants. The experiment results are presented in Figure 6, where it can be observed that rapid nutrient release occurs within the first 4 h, followed by a stabilization of the release rate up to 72 h. This phenomenon may be attributed to establishing an initial equilibrium in the tested system and soil saturation, which slows down the release rate, indicating that fresh composites exhibit controlled-release properties of fertilizer substances [30]. Subsequently, after 96 h, a linear decline in nutrient release is observed, reaching a near-complete end after 168 h. The achievement of controlled release is likely the result of electrostatic interactions between alginate and chitosan [31]. Considering the results of the swelling tests, dried structures lose their functional properties due to excessive water loss. In contrast, fresh structures continue to exhibit the behavior described in the literature for the interactions of the applied biopolymers. The initial rapid release of nutrients may result from ion exchange with soil-contained ions, which initially intensifies the release process [32]. The eventual attainment of equilibrium confirms this.

#### 3.1.3. Biodegradability

**Figure 7:**
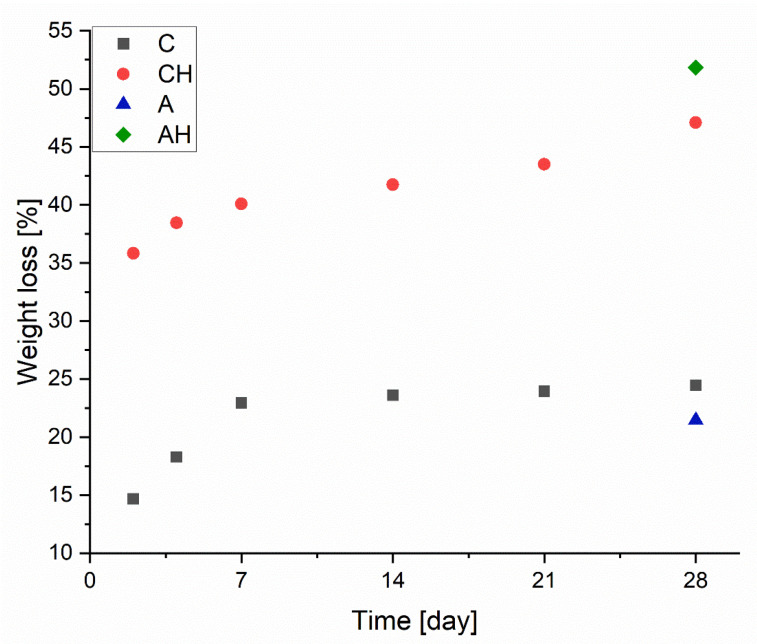
Mass change during the biodegradation process.

Biodegradation tests were conducted on dried composites over 4 weeks to evaluate mass loss under less invasive conditions than column leaching. Figure 7 presents the degree of degradation, where matrices without hydrolysate (C) initially exhibit a linear mass loss, stabilizing at approximately 23%. After the degradation process, this value reaches around 24.5%, showing minimal change despite the process continuing for an additional 3 weeks. This result indicates the high stability of the obtained encapsulated structures.

For comparison, the group without the chitosan coating (A) achieved a similar degree of biodegradation after 28 days, approximately 22%. In the case of composites with the addition of hydrolysate, a significant increase in degradation is observed, with no stabilization in degradation rates during the experiment. The mass loss progresses linearly from 36% after 2 days to 47% after 48 days, contrasting with sample C’s results. This discrepancy arises from the interaction of hydrolysate, with its slightly acidic pH, with alginate and chitosan, increasing their biodegradability and reducing their stability, leading to substantial mass loss [33]. However, it is worth noting that for the composite containing hydrolysate without a chitosan coating (AH), a significant mass loss of 52% was recorded. This demonstrates that incorporating the chitosan coating diminished the degradation rate of the composite, aligning with the existing literature on sodium alginate and chitosan interactions [34].

### 3.2. Morphology

#### 3.2.1. XRD Analysis

**Figure 8:**
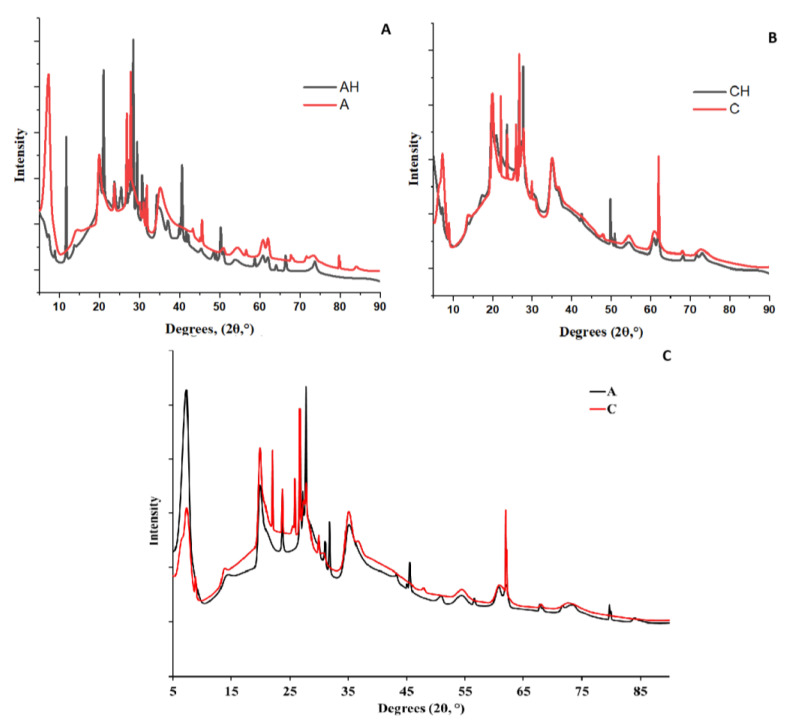
Compilation of XRD analyses of composites, ((**A**)—comparison of AH and A XRD analysis; (**B**)—comparison of CH and C XRD analysis; (**C**)—comparison of A and C XRD analysis).

An XRD analysis was conducted to identify structural differences in the obtained composites. In Figure 8A, a comparison between matrices A and AH is presented. In sample A, which represents the base matrix without adding hydrolysate or a chitosan coating, sharp peaks are observed in the low-angle range of 10–40°, indicating the presence of a crystalline structure [35]. This structure is more porous and less homogeneous, consistent with results from leaching studies. The crystallinity of the sample is evidenced by prominent peaks in the 5–20° range, which are reduced in intensity for the AH matrix containing hydrolysate, leading to increased amorphousness and structural homogeneity. This is likely due to interactions between the hydrolysate and alginate and the protonation of -COOH groups, which weakens crosslinking and reduces structural stability, a phenomenon also confirmed by biodegradation studies [36,37].

In the comparison between composites A and C (Figure 8C), it can be observed that the addition of the chitosan coating, similar to the hydrolysate, increased the amorphous nature of the structure and enhanced its densification. This is attributed to hydrogen and amide bonds forming between sodium alginate and chitosan [38]. Additionally, chitosan reacts with bentonite, forming microcrystalline regions in the 15–30° range, characteristic of aluminosilicates [39].

Figure 8B shows that the addition of hydrolysate further weakens the intensity of the crystalline structure in the 15–30° range, which can be attributed to the degradation of bentonite, known for its susceptibility to acidic environments. The hydrolysate appears to further increase the amorphous nature of the structure, not only through the protonation of -COOH groups as observed in the AH group, but also through the protonation of amino groups (-NH_3_^+^) present in chitosan [31,39]. This results in a synergistic effect, increasing the number of hydrogen bonds and ionic interactions between chitosan and sodium alginate. The final structure is characterized by the lowest porosity and the highest homogeneity, as confirmed by leaching and swelling tests. The mass increase due to swelling was the weakest in the CH group.

#### 3.2.2. TGA Analysis

**Figure 9:**
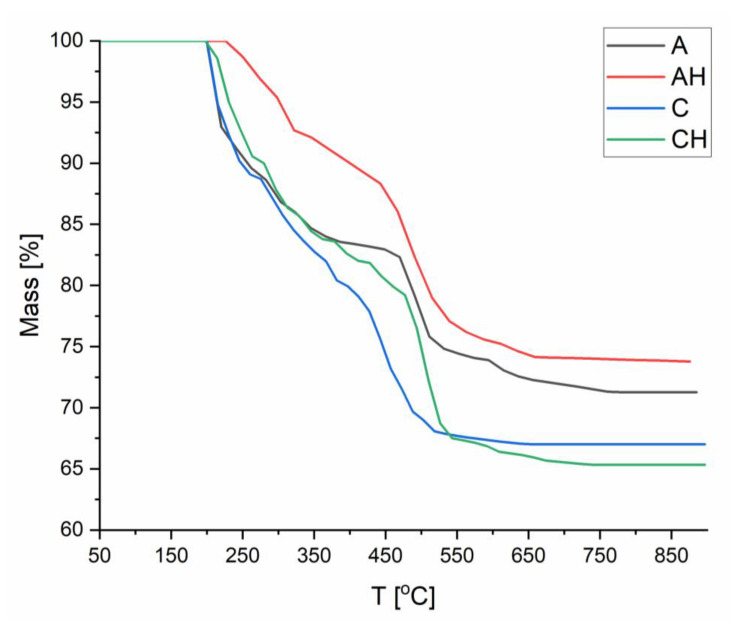
Compilation of TGA analyses of composites.

A TGA analysis was conducted to determine the impact of hydrolysate addition and chitosan coating on the thermal stability of the obtained composites. Analyzing Figure 9, which compares matrices A and AH, it can be observed that in the initial stage (up to 150 °C), no degradation occurs due to dried samples (absence of residual moisture). After exceeding 150 °C, up to approximately 300 °C, depolymerization of sodium alginate occurs, associated with the degradation of hydroxyl (-OH) and carboxyl (-COOH) groups [40]. Notably, adding hydrolysate in sample AH reduced the degree of degradation, which may be related to the formation of additional hydrogen bonds resulting from the protonation of carboxyl groups [41]. This phenomenon was confirmed in the XRD analysis and led to a shift in the degradation of polymer chains to higher temperatures. In the 300–500 °C range, further degradation of sodium alginate occurs; however, an amorphous structure is obtained due to the addition of hydrolysate, facilitated by the presence of K⁺ and SO_4_^2−^ ions, stabilizing sodium alginate. The reduced porosity further delays the degradation process [42,43]. At later stages, the degradation curves appear similar, and the final differences may result from the increased amount of material in sample AH, associated with the hydrolysate addition.

In Figure 9, a comparison between matrices A and C is presented. Up to 350 °C, no significant differences in thermal degradation are observed between the two composites. However, beyond this temperature, faster degradation occurs in sample C due to the lower thermal resistance of chitosan, where amide groups and polymer chains undergo decomposition [44]. The formation of ionic bonds between chitosan and alginate increases the amorphous nature of the structure, which in turn reduces the thermal strength of the composite, leading to a faster degradation rate compared to sample A. Chitosan does not form stable carbonaceous residues, which results in a lower final residual mass.

Finally, Figure 9 compares the structures of C and CH, where thermal degradation proceeds almost identically. The difference occurs in the temperature range of 400–500 °C, where the addition of hydrolysate in group CH increases thermal stability. This effect may result from the protonation of -COOH groups, which strengthens hydrogen bonds and promotes ionic interactions with hydrolysate ions (K⁺ and SO_4_^2−^) [38,42,43]. These bonds delay the degradation process. However, at higher temperatures, hydrolysate ions may act as oxidation catalysts, increasing the degree of degradation. The lower residual content is also attributed to the higher amorphous nature of the CH structure, which makes it more susceptible to oxidation processes. The study demonstrated that adding hydrolysate can positively influence biopolymeric structures by forming bonds in the temperature range of 300–600 °C. However, at higher temperatures, it can catalyze the oxidation process.

#### 3.2.3. FTIR Analysis

**Figure 10:**
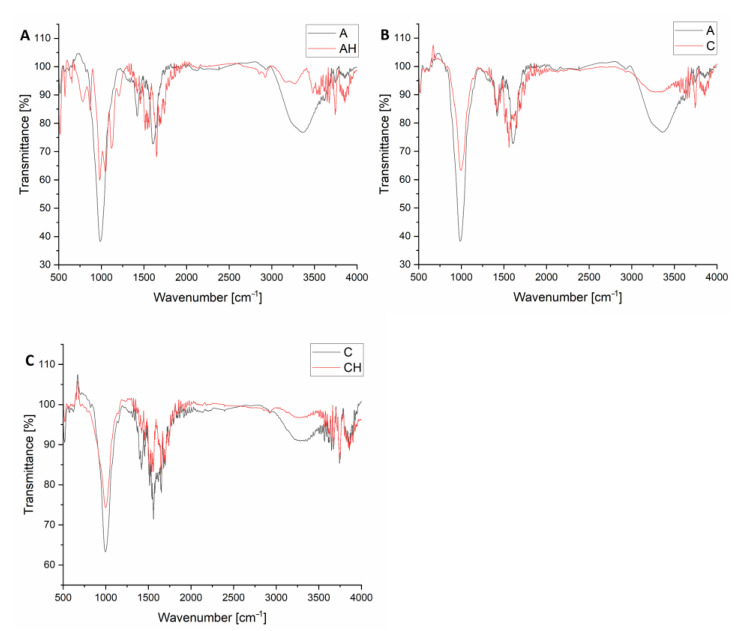
Compilation of FTIR analyses of composites, ((**A**)—comparison of A and AH group FTIR analysis; (**B**)—comparison of A and C group FTIR analysis; (**C**)— comparison of C and CH group FTIR analysis).

In Figure 10A, when comparing the spectra of matrices A and AH, it can be observed that the introduction of hydrolysate into the AH matrix leads to several significant changes. In the 3000–3500 cm^−1^ range, the band corresponding to O-H and N-H group vibrations becomes more intense and broader, indicating the presence of additional hydroxyl groups from glycerol and amino groups from amino acids [45]. This serves as direct evidence of the incorporation of protein and fat hydrolysis products into the microparticle structure [46]. Another notable signal is the band at 1655 cm^−1^, which appears to be more prominent in the AH matrix than in that of A. This band can be attributed to the stretching vibrations of C=O groups from carbonyl groups present in amino acids, confirming the presence of protein hydrolysis products [47]. This band is weaker in matrix A, suggesting that additional amino acid sources are absent. An increased intensity of bands can be observed in the AH matrix in the range of 1150–1075 cm^−1^, associated with C-N vibrations. These bands confirm the presence of amino acids and aliphatic amines, key products of protein hydrolysis in the hydrolysate. Similarly, the band at 940 cm^−1^, which is not visible in matrix A, becomes distinct in matrix AH. The introduction of hydrolysate also affects the carboxyl groups of sodium alginate. In the AH matrix, broadening and shifting of the 1600–1550 cm^−1^ band (C=O bonds from carboxylate groups) are observed, suggesting the protonation of carboxyl groups due to the hydrolysate’s low pH [48]. This protonation strengthens hydrogen bonding interactions with amino acids and glycerol in the hydrolysate, stabilizing the matrix structure and reducing the number of free carboxylate groups [41]. Glycerol is most likely formed due to the hydrolysis of fat contained in the larvae, which is a common phenomenon, as the triacylglycerols present in the larvae are broken down into glycerol and fatty acids [49]. Additionally, in the range of 1150–1000 cm^−1^, bands characteristic of C-O-C and Si-O-Si vibrations weaken and become broader in the AH matrix [50]. This is likely the result of the hydrolysate’s acidic environment interacting with bentonite’s layered structure, leading to partial modification or degradation of its crystalline structure. These changes confirm that the hydrolysate introduces protein and fat hydrolysis products into the matrix, influencing its chemical stability, reducing porosity, and increasing the amorphous nature of the AH structure. The FTIR results are consistent with TGA analyses (improved thermal stability) and XRD analyses (greater amorphousness of the structure).

The comparison of FTIR spectra for matrices A and C, presented in Figure 10B, reveals significant yet subtle differences resulting from the 2% chitosan in matrix C. Both matrices have an almost identical composition and were prepared using the same methods, except for the additional chitosan component in matrix C. Despite this minor compositional difference, the FTIR analysis indicates the appearance of characteristic peaks and modifications in the existing bands. For matrix C, containing 2% chitosan, additional peaks appear at 2880 cm^−1^ and approximately 749 cm^−1^, either absent or much weaker in the spectrum of matrix A [51]. The band at 2880 cm^−1^ corresponds to the C-H stretching vibrations in aliphatic chains of chitosan polysaccharides. This is typical for methyl and methylene groups present in the chitosan structure and confirms the successful incorporation of this component into matrix C [52]. Meanwhile, the signal at 749 cm^−1^ corresponds to the C-H deformation, characteristic of polysaccharides such as chitosan. In addition to the new peaks, introducing chitosan also influences existing signals in matrix A, resulting from interactions between matrix components and chitosan’s functional groups. In the 3400–3200 cm^−1^ range, broadening and increased intensity of the band corresponding to O-H stretching vibrations are observed. This change arises from forming hydrogen bonds between the hydroxyl and amino groups of chitosan and the carboxylate groups (-COO^−^) of sodium alginate. As a polycationic polysaccharide, chitosan interacts with the anionic carboxylate groups of alginate, enhancing the structural stability of the microparticles [53]. In the 1600–1550 cm^−1^ range, corresponding to C=O stretching vibrations in carboxylate groups of sodium alginate, band broadening and shifting are visible in matrix C. This effect results from forming amide bonds between the amino groups (-NH_2_) of chitosan and the carboxylate groups (-COO^−^) of alginate. These reactions confirm that chitosan not only fills the matrix structure but also chemically interacts with it, modifying its properties. In the 1150–1000 cm^−1^ range, characteristic of C-O-C vibrations in starch and Si-O-Si vibrations in bentonite, a reduction in band intensity is observed in matrix C [50]. Chitosan introduces additional bonds and structural disruptions, reducing the organization of existing matrix components such as starch and bentonite. As a result, an increase in amorphousness is observed, consistent with the XRD analysis, which revealed weakened crystalline peaks in matrix C.

The comparison of FTIR spectra for matrices C and CH (Figure 10C) reveals significant differences arising from the introduction of hydrolysate into the microparticle structure containing 2% chitosan. Hydrolysate at pH 5.0 significantly modifies the spectrum due to amino acids, glycerol, and fat hydrolysis products interacting with the existing matrix components [49]. The most notable effect is the broadening and increased intensity of the band in the 3400–3200 cm^−1^ range, corresponding to the stretching vibrations of hydroxyl (-OH) and amino (-NH_2_) groups [54]. This band is more pronounced in matrix CH than in matrix C, likely due to glycerol and amino acids from the hydrolysate [45,55]. These groups form hydrogen bonds with chitosan and sodium alginate, enhancing the matrix’s chemical stability and structural uniformity. The increased signal intensity indicates more active functional groups being introduced into the matrix with the hydrolysate. Another significant difference is the enhancement of the band around 1655 cm^−1^, corresponding to the C=O stretching vibrations from carbonyl groups present in amino acids, the main products of protein hydrolysis. In the spectrum of matrix CH, this signal is much more intense than in matrix C, confirming the presence of amino acids within the microparticle structure [56]. In matrix C, which lacks hydrolysate, this band is weaker and originates solely from the carbonyl groups of alginate and other base components. In the 940 cm^−1^ range, an additional band appears in matrix CH, absent in matrix C. This band corresponds to C-H vibrations in alkane chains derived from fat hydrolysis products in the hydrolysate [46]. In the 1150–1000 cm^−1^ range, corresponding to C-O-C (starch) and Si-O-Si (bentonite) stretching vibrations, the bands weaken and broaden in matrix CH [50]. The hydrolysate disrupts the crystalline structures of starch and bentonite. This leads to a decreased structural order and an increased amorphous phase, consistent with the XRD analysis results.

### 3.3. Germination Tests

**Figure 11:**
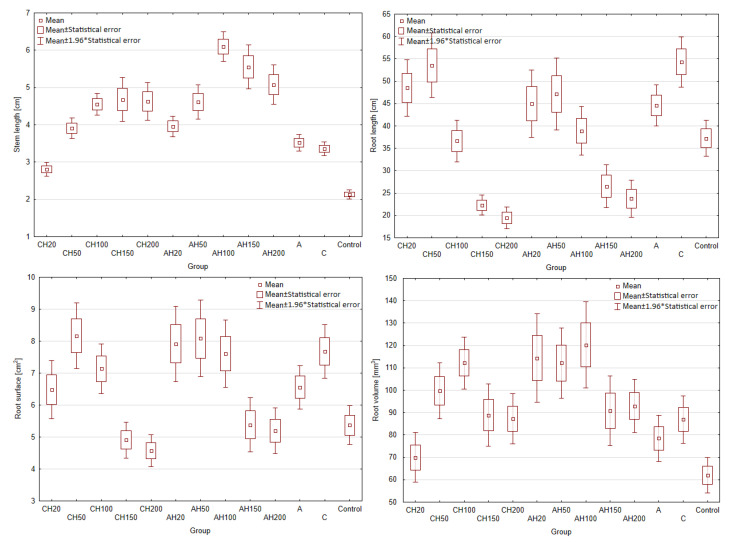
Biometric parameters of germination tests, (* indicates *p* < 0.05 compared to the control group).

Germination tests were conducted to determine the phytotoxic dose of the hydrogel matrices, as hydrolysates demonstrated phytotoxic effects at doses of 20% and 50%. Additionally, it is essential to assess the impact of encapsulated matrices on plant growth compared to non-encapsulated ones, as this should indicate changes in the nutrient release rate. Due to the gradual nutrient release properties, doses of 20%, 50%, 100%, 150%, and 200% were tested for AH and CH media. A total of 13 fertilizer groups were evaluated, along with three control groups: Control—without any fertilizing compounds; group A—supplemented with biopolymer capsules containing only distilled water and matrix additives; and group C—containing matrix components and a chitosan coating. For comparison, the number of capsules in groups A and C was equal to that used for the 100% dose of those containing hydrolysates. The fertilizer dose was calculated based on the nitrogen requirement of the test plant, field cucumber (140 kg/ha). To ensure comparability of the fertilizing effect, the number of capsules was standardized due to minor differences in nitrogen content between the two capsule types. The results of the germination tests are presented in Figure 11 and Table 1.

As a result of the research, no phytotoxic effect was observed in any of the tested groups, even when applying a 200% dose. This indicates a slow-release effect and improved plant supplementation; in the study by [5,6], a phytotoxic effect occurred at 20% and 50% hydrolysate doses. Comparing the stem growth shown in Figure 11, it can be observed that for the AH matrices, there is a linear increase up to the 100% dose, followed by a decline at 150% and 200%, which may indicate over-fertilization and excessively rapid nutrient release during the early stages of plant development.

For the encapsulated CH composites, similar linear growth is noted with increasing doses; however, after exceeding the 100% dose, growth stagnates, with results aligning closely to the AH50 dose. This may result from limited nutrient exchange between the CH matrix and the environment, as well as the lower ability of these capsules to re-release and retain water, as confirmed in the swelling tests. Nevertheless, a 25% increase in stem growth was recorded for the AH matrices compared to the CH matrices, proving that the encapsulation limits exchange with the environment. However, this could also be attributed to the loss of hydrolysate during the encapsulation process. This hypothesis is further supported by the observation that the CH20 group exhibited significantly poorer growth than control groups C and A, which showed similar growth levels. This result is particularly unexpected, as nitrogen groups within the chitosan coating should be accessible to plants and promote their growth.

Analyzing root growth parameters further reveals a similar trend for both composites. Lower doses of biopolymers (20% and 50%) promote better root development than higher fertilizer doses, which is likely related to hydrolysate. This conclusion is supported by the fact that control groups without hydrolysate showed similar growth patterns, with group C exhibiting better root development than group A, most likely due to the nitrogen content in the chitosan. Once again, it can be deduced that CH groups exhibit reduced nutrient release due to restricted exchange with the environment, as seen in groups CH20 and CH50. Although, at first glance, groups CH100, CH150, and CH200 may appear to contradict this observation, their results fall within the margin of statistical error.

**Figure 12:**
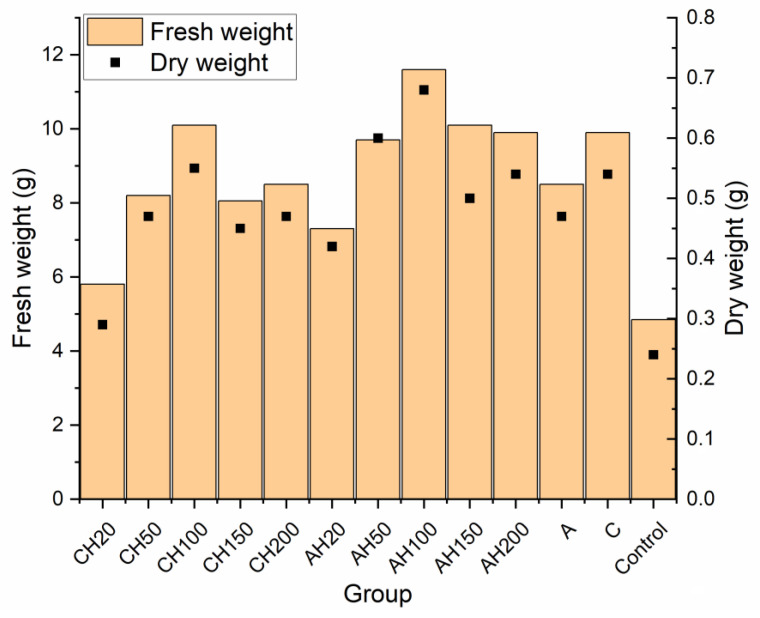
Comparative analysis of fresh and dry weights in germination tests.

A similar trend is visible in the root surface area and volume, confirming the previously drawn conclusions. Figure 12 presents a summary of the biomass of the cultivated plants. Analyzing the graph, it is evident that adding the chitosan coating significantly reduced the effectiveness of the biopolymeric fertilizer on plant development, even when compared to the control groups. Comparing the 100% dose, which corresponds to the number of capsules used in control groups A and C, it becomes clear that the change in fresh and dry biomass is negligible, which cannot be said for group AH100. This indicates that while the chitosan coating effectively limited nutrient exchange with the environment, it also caused the fertilizer to lose many beneficial properties. The poorer performance of the 20% groups compared to the control groups A and C can be attributed to the fact that both alginate matrices, with and without coatings, were used in quantities equal to the 100% dose. Alginate is commonly employed as a seed germination aid due to its ability to regulate water management in plants. Similarly, chitosan has been shown in various studies to positively impact plant growth by increasing their size, biomass, and chlorophyll content, indicating its beneficial effect on plants [57,58]. This demonstrates that the application of these biopolymers leads to improved plant productivity compared to the Control group without any fertilization and groups below 100%, where the content of the biopolymers was lower. These conclusions are evident when comparing the results presented in Figure 11 and Figure 12.

### 3.4. Future Perspective

The current research presents several limitations, primarily related to the restricted scope of examinations, particularly in the in vivo tests and biodegradability analysis performed over a short timeframe. The aim was to identify trends, the potential of the proposed solution, and the limitations associated with the presented method. The research demonstrated that the proposed method’s main challenge is the AH matrix’s high porosity. It leads to the rapid release of the encapsulated hydrolysate, limiting its applicability. As confirmed by morphological studies, chitosan coatings were incorporated to reduce porosity. However, the hydrolysate encapsulated in the AH matrix was released into the chitosan solution during the coating process, which suggests that the coating process is too slow or that the bonding between oppositely charged biopolymers occurs at an inadequate rate.

It is recommended that the research scope be extended to include other matrix additives for the AH matrix. For example, glycerol is suggested as a potential solution, given its role as a plasticizer that interacts with hydroxyl groups, increasing mobility and enhancing crosslinking capabilities with calcium ions (Ca^2^⁺). It is also possible to add hydrolysate during the encapsulation and crosslinking of alginate to increase its concentration, thereby reducing the intensity of diffusion processes. Adjusting the pH of the mixture during the crosslinking process is proposed to stabilize the sodium alginate structure and facilitate the formation of bonds between the cationic groups of chitosan and the anionic groups of alginate. In this context, optimizing the pH of the AH hydrolysate encapsulated within the matrix may also prove beneficial.

It is also worth considering the use of higher chitosan concentrations, such as 3% *w*/*w*, as the increased number of amino groups could contribute to the faster formation of more stable structures. According to the available literature, alginate reacts more rapidly with chitosan at lower temperatures (3–10 °C), which could be explored further alongside the introduction of additional crosslinking agents, such as Ca^2^⁺ ions. Alternatively, modifying the structure of the alginate matrix by adding glycerol may enhance matrix stabilization and reduce the initial release of nutrients.

A key step in verifying these recommendations would be conducting a balanced analysis, quantifying the hydrolysate released at each production stage. Long-term tests should be performed only after addressing these identified challenges, including 60-day biodegradability studies and 30-day soil pot tests using multi-pot setups in controlled environments. These tests will help determine whether the proposed solution enables sustained nutrient release and plant nutrient uptake over the long term.

## 4. Conclusions

This study demonstrates that biopolymer matrices are a practical solution for encapsulating liquid fertilizers. They allow for the application of higher doses without causing phytotoxic effects in plants. This technology addresses two critical challenges in modern agriculture, which are over-fertilization and excessive water consumption. The research outlines key areas for improvement, identifies limitations, and highlights challenges that must be addressed to ensure its applicability in controlled environments and industrial applications.

The novelty of this study comes from fully biodegradable composites being utilized for encapsulating liquid fertilizers, a topic not previously explored in the literature. Utilizing biopolymers’ unique properties, this method offers a sustainable alternative to conventional fertilizers, reducing nutrient leaching, environmental contamination, and resource inefficiency. These composites have significant potential in greenhouse farming, ensuring precise nutrient delivery while serving as a water storage system. In hydroponic systems, biopolymer matrices can prevent the accumulation of salts, maintain water quality, and reduce the need for frequent purification.

This research provides a novel pathway for integrating biopolymer technology into agricultural systems, particularly in regions with water shortages and degraded soils. It offers a strong starting point for future studies and lays the groundwork for addressing critical challenges in sustainable agriculture, paving the way for innovative and eco-friendly farming solutions.

## Figures and Tables

**Table 1:** Mean plant growth parameters for germination tests.

Group	Stem Length [cm]	Root Length [cm]	Root Area [cm^2^]	Root Volume [mm^3^]
CH20	2.81 ± 0.47 ^abcdefghij^	48.51 ± 16.20 ^abcd^	6.48 ± 2.31	69.96 ± 28.35 ^abcd^
CH50	3.90 ± 0.70 ^aklmn^	53.58 ± 18.49 ^efghijk^	8.17 ± 2.61 ^abcde^	99.80 ± 31.76 ^e^
CH100	4.55 ± 0.75 ^bopqrs^	36.63 ± 12.04 ^elmno^	7.14 ± 1.97 ^fg^	112.16 ± 29.40 ^afg^
CH150	4.68 ± 1.50 ^ctuvw^	22.30 ± 5.74 ^aflpqrstu^	4.91 ± 1.43 ^afhijk^	88.92 ± 35.59
CH200	4.63 ± 1.28 ^dxyzAB^	19.47 ± 6.13 ^bgmvwxyzA^	4.57 ± 1.27 ^bglmnop^	87.24 ± 28.45 ^h^
AH20	3.96 ± 0.64 ^eCDEF^	44.98 ± 17.60 ^pvBC^	7.92 ± 2.76 ^hlqrs^	114.33 ± 46.21 ^bij^
AH50	4.61 ± 1.07 ^fGHIJ^	47.17 ± 18.74 ^qwDE^	8.09 ± 2.80 ^imtuv^	112.14 ± 36.80 ^ckl^
AH100	6.09 ± 0.88 ^gkotxCGKLMN^	38.92 ± 12.08 ^hrxFG^	7.61 ± 2.34 ^jnwxy^	120.21 ± 42.81 ^dhmno^
AH150	5.55 ± 1.47 ^hlpyDOPQ^	26.53 ± 12.00 ^ciBDHI^	5.38 ± 2.12 ^cqtwz^	90.79 ± 38.96
AH200	5.08 ± 1.30 ^imEKRST^	23.75 ± 10.38 ^djnCEFJKL^	5.19 ± 1.77 ^druxA^	93.00 ± 29.76 ^p^
A	3.52 ± 0.56 ^jquzHLORU^	44.60 ± 11.44 ^syHJ^	6.56 ± 1.70 ^o^	78.46 ± 26.07 ^fikm^
C	3.36 ± 0.45 ^rvAIMPSV^	54.30 ± 14.29 ^otzGIKM^	7.68 ± 2.13 ^kpzAB^	86.96 ± 27.06 ^n^
Control	2.13 ± 0.31 ^nswBFJNQTUV^	37.26 ± 10.38 ^kuALM^	5.38 ± 1.56 ^esvyB^	62.04 ± 19.99 ^egjlop^

a(A),b(B),c(C),…—results marked with the same letter differ significantly statistically—*t*-test (*p* < 0.05), vertical comparison.

## Data Availability

The original contributions presented in this study are included in the article. Further inquiries can be directed to the corresponding author.
